# Enhanced production of γ-amino acid 3-amino-4-hydroxybenzoic acid by recombinant *Corynebacterium glutamicum* under oxygen limitation

**DOI:** 10.1186/s12934-021-01714-z

**Published:** 2021-12-23

**Authors:** Hideo Kawaguchi, Tomohisa Hasunuma, Yasuo Ohnishi, Takashi Sazuka, Akihiko Kondo, Chiaki Ogino

**Affiliations:** 1 Graduate School of Science, Technology and Innovation, Kobe University, 1-1 Rokkodai, Nada, Kobe, 657-8501 Japan; 2 Engineering Biology Research Center, Kobe University, 1-1 Rokkodai, Nada, Kobe, 657-8501 Japan; 3 Department of Biotechnology, Graduate School of Agricultural and Life Sciences, The University of Tokyo, 1-1-1, Yayoi, Bunkyo, Tokyo, 113-8657 Japan; 4 Collaborative Research Institute for Innovative Microbiology, The University of Tokyo, Bunkyo, Tokyo, 113-8657 Japan; 5 Bioscience and Biotechnology Center, Nagoya University, Furo, Chikusa, Nagoya, 464-8601 Japan; 6 Department of Chemical Science and Engineering, Graduate School of Engineering, Kobe University, 1-1 Rokkodai, Nada, Kobe, 657-8501 Japan; 7 Biomass Engineering Research Division, RIKEN, 1-7-22 Suehiro, Tsurumi, Yokohama, Kanagawa 230-0045 Japan

**Keywords:** *Corynebacterium glutamicum*, Dissolved oxygen, Metabolic engineering, Metabolome analysis, Amino acid

## Abstract

**Background:**

Bio-based aromatic compounds are of great interest to the industry, as commercial production of aromatic compounds depends exclusively on the unsustainable use of fossil resources or extraction from plant resources. γ-amino acid 3-amino-4-hydroxybenzoic acid (3,4-AHBA) serves as a precursor for thermostable bioplastics.

**Results:**

Under aerobic conditions, a recombinant *Corynebacterium glutamicum* strain KT01 expressing *griH* and *griI* genes derived from *Streptomyces griseus* produced 3,4-AHBA with large amounts of amino acids as by-products. The specific productivity of 3,4-AHBA increased with decreasing levels of dissolved oxygen (DO) and was eightfold higher under oxygen limitation (DO = 0 ppm) than under aerobic conditions (DO ≥ 2.6 ppm). Metabolic profiles during 3,4-AHBA production were compared at three different DO levels (0, 2.6, and 5.3 ppm) using the DO-stat method. Results of the metabolome analysis revealed metabolic shifts in both the central metabolic pathway and amino acid metabolism at a DO of < 33% saturated oxygen. Based on this metabolome analysis, metabolic pathways were rationally designed for oxygen limitation. An *ldh* deletion mutant, with the loss of lactate dehydrogenase, exhibited 3.7-fold higher specific productivity of 3,4-AHBA at DO = 0 ppm as compared to the parent strain KT01 and produced 5.6 g/L 3,4-AHBA in a glucose fed-batch culture.

**Conclusions:**

Our results revealed changes in the metabolic state in response to DO concentration and provided insights into oxygen supply during fermentation and the rational design of metabolic pathways for improved production of related amino acids and their derivatives.

**Graphical Abstract:**

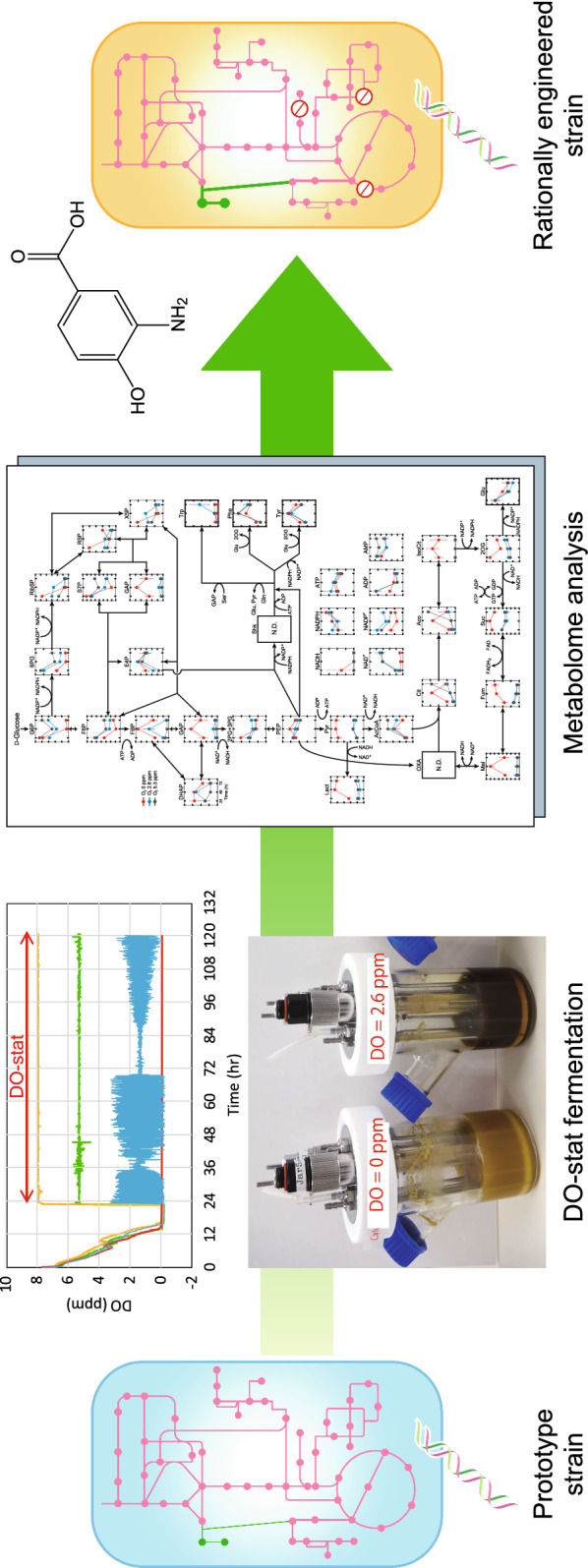

**Supplementary Information:**

The online version contains supplementary material available at 10.1186/s12934-021-01714-z.

## Background

Bio-based aromatic compounds are of great interest to the industry as commercial production of aromatic compounds depends exclusively on the unsustainable use of fossil resources or extraction from plant resources [1]. Because aromatic compounds are essential precursors for the synthesis of strong, thermostable plastics [2, 3], metabolic engineering of the shikimate pathway for microbial production of aromatic compounds, such as caffeic acid [4], (*S*)-reticuline [5], salicylate [6], and styrene [7], has been studied since the 2000s. More recently, 3-amino-4-hydroxybenzoic acid (3,4-AHBA), which serves as a precursor for ultra-thermoresistant bioplastics, has been produced from lignocellulosic biomass [8].

**Fig. 1:**
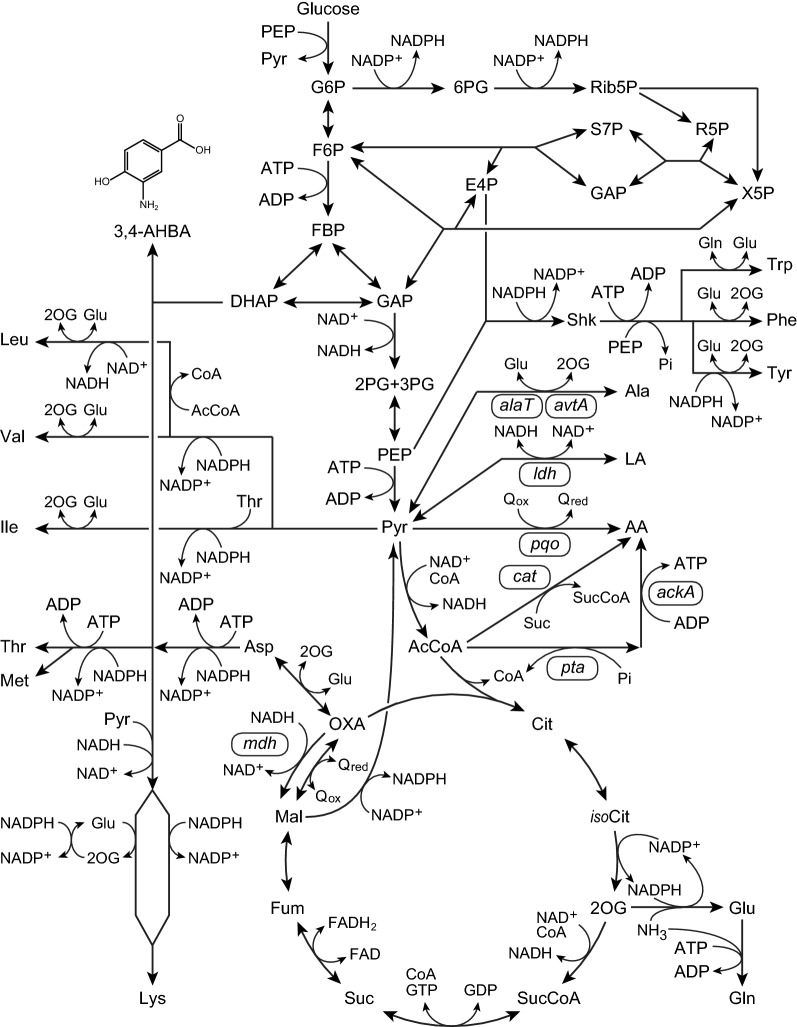
Metabolic pathway for the production of 3-amino-4-hydroxybenzoic acid (3,4-AHBA) and related metabolites. Genes deleted from the chromosome of *C. glutamicum* strain KT01 are indicated in rounded rectangles

The synthetic pathway of γ-amino acid 3,4-AHBA differs entirely from the well-known shikimate pathway, which is essential for synthesizing aromatic amino acids, l-phenylalanine (Phe), l-tyrosine (Tyr), and l-tryptophan (Trp) [9]. In a simple two-step reaction (aldol condensation followed by cyclization and aromatization), an aromatic ring of 3,4-AHBA is formed from C_4_ (l-aspartate-4-semialdehyde) and C_3_ (dihydroxyacetone phosphate [DHAP]) primary metabolites. In *Streptomyces griseus*, the enzymes GriI and GriH, encoded by two genes, *griI* and *griH*, respectively, catalyze 3,4-AHBA synthesis [10]. A recombinant *Corynebacterium glutamicum* strain expressing the *griH* and *griI* genes successfully produced 3,4-AHBA from sugars [11] (Fig. 1). However, strategies to improve 3,4-AHBA production, including metabolic engineering and bioengineering, need to be identified.

Dissolved oxygen (DO) concentration is one of the most important factors determining the performance of the fermentation process [12]. Unlike anaerobic fermentation of organic acids, the formation of aromatic compounds and amino acids requires oxygen, as reduced nicotinamide adenine dinucleotide phosphate (NADPH) required for the synthesis is primarily supplied through the oxidative pentose phosphate pathway (PPP; Fig. 1) [13, 14]. For example, the production of aspartate derivatives of l-lysine (Lys) and 1,5-diaminopentane was implemented at a DO level of ≥ 20% saturated oxygen to regenerate NADPH [15–17]. In addition, aromatic compounds of shikimate and its derivatives have been produced at a DO of ≥ 10% saturated oxygen level in culture using *E. coli* and *C. glutamicum* [18, 19].

3,4-AHBA production competes with Lys biosynthesis for the availability of the common precursor l-aspartate-4-semialdehyde derived from aspartate. In glucose metabolism, 2 mol of NADPH is required to synthesize 1 mol of 3,4-AHBA, while 4 mol of NADPH is required to synthesize 1 mol of Lys [16]. In *C. glutamicum*, NADPH supply predominantly depends on oxidative PPP, and the increased flux improves Lys yield (Fig. 1) [15, 20]. These suggest that a diminished NAPDH supply under oxygen restriction can direct more carbon from Lys to 3,4-AHBA. However, metabolic profiles, including amino acid synthesis under oxygen-limited conditions, have not been extensively studied, although the transcriptome of *C. glutamicum* has been extensively studied under different DO conditions [12, 21–23].

The present study demonstrated enhanced 3,4-AHBA production by recombinant *C. glutamicum* under oxygen limitation in a jar fermentor using the DO-stat program. Using the DO-stat method, metabolic profiles at three different levels of DO were compared, and the metabolome analysis revealed metabolic shifts in both the central metabolic pathway and amino acid metabolism at a threshold DO level. Based on the metabolome analysis results, the metabolic pathway of *C. glutamicum* was rationally designed to tailor to oxygen limitation and thus significantly improve the specific productivity of 3,4-AHBA.

## Results

### Enhanced 3,4-AHBA production under low DO concentration

**Fig. 2:**
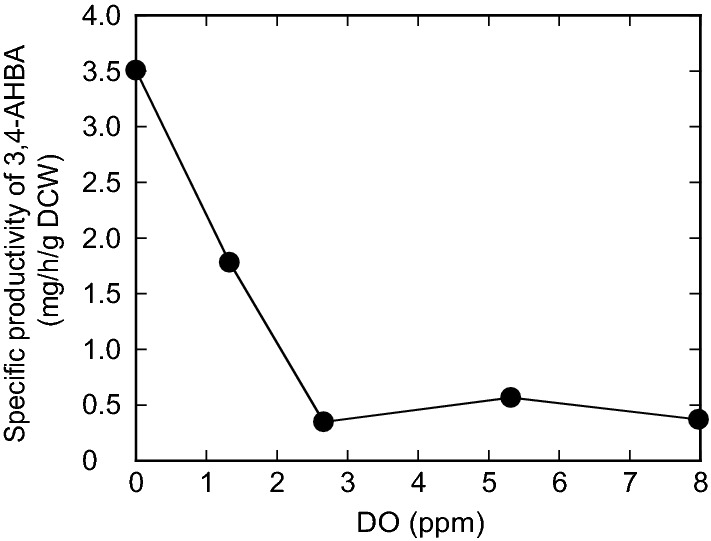
Effect of dissolved oxygen (DO) concentration on the specific productivity of 3-amino-4-hydroxybenzoic acid (3,4-AHBA). The specific productivity of 3,4-AHBA in recombinant *C. glutamicum* strain KT01 after 72 h of cultivation is indicated. The DO-stat method was started after 24 h of cultivation, and five DO levels (0, 1.3, 2.6, 5.3, and 8.0 ppm) were implemented and controlled by the speed of agitation. The data are presented as averages ± standard deviation calculated from the results of duplicate independent experiments

To examine the effect of DO on 3,4-AHBA production from glucose by recombinant *C. glutamicum*, the DO-stat method was implemented to control DO levels in culture medium at 1.3, 2.6, 5.3, and 8.0 ppm (representing the range from 17 to 100% saturated oxygen concentration) after 24 h of cultivation. In culture at a DO level of 0 ppm (representing 0% saturated oxygen concentration), the agitation speed was fixed at 200 rpm to ensure that the oxygen supply was the rate-limiting step for *C. glutamicum* cells to produce 3,4-AHBA. After 72 h of cultivation, the specific productivity of 3,4-AHBA showed a negative correlation with the DO level (Fig. 2). The specific productivity of 3,4-AHBA was comparable (0.4 mg 3,4-AHBA/h/g dry cell weight [DCW]) at high DO levels (≥ 2.6 ppm), while it sharply increased with reduced DO levels ≤ 1.3 ppm, reaching 3.5 mg /h/g DCW at a DO level of 0 ppm.

**Fig. 3:**
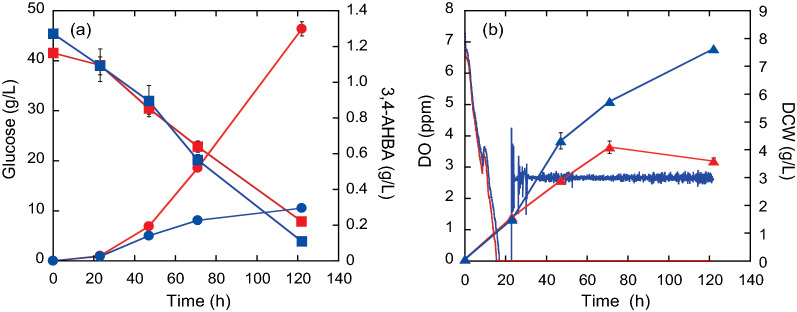
Dissolved oxygen (DO)-stat cultivation for 3-amino-4-hydroxybenzoic acid (3,4-AHBA) production by recombinant *C. glutamicum* strain KT01. **a** The concentrations of glucose (squares), 3,4-AHBA (circles), **b** dry cell weight (DCW) (triangles), and DO concentration (continuous lines) are indicated. The DO-stat method was started after 24 h of cultivation, and two DO levels (0 [reds] and 2.6 ppm [blues]) were implemented and controlled by the speed of agitation. The data are presented as averages ± standard deviation calculated from the results of duplicate independent experiments

Compared to a high DO level (2.6 ppm), cell growth was reduced by 44% after 72 h of cultivation under a low DO level of 0 ppm, whereas the glucose consumption rate was comparable throughout the period of 122 h of cultivation (Fig. 3). Conversely, 3,4-AHBA concentration after 122 h of cultivation was 4.4-fold higher at a DO level of 0 ppm than at 2.6 ppm. These results suggest that oxygen limitation enhances 3,4-AHBA production, probably by directing more carbon and nitrogen from cell growth to 3,4-AHBA production.

### Metabolic shift from amino acids to organic acids under low DO conditions

**Fig. 4:**
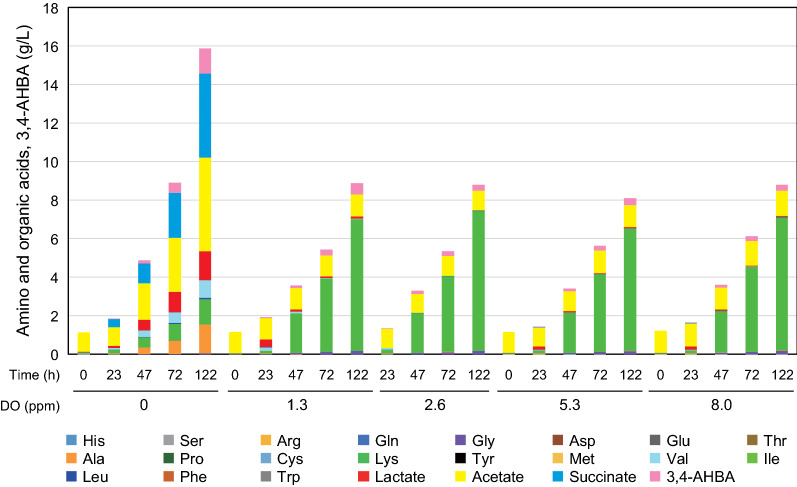
Effect of dissolved oxygen (DO) concentration on the production of amino acids and organic acids. The DO-stat method was started after 24 h of cultivation, and five DO levels (0, 1.3, 2.6, 5.3, and 8.0 ppm) were implemented and controlled by the speed of agitation during 3-amino-4-hydroxybenzoic acid (3,4-AHBA) production by recombinant *C. glutamicum* strain KT01. The data are presented as averages calculated from the results of duplicate independent experiments

To investigate enhanced 3,4-AHBA production under oxygen limitation, extracellular concentrations of amino acids and organic acids during 3,4-AHBA production were determined. At DO levels ≥ 1.3 ppm, the composition and concentration of external amino acids were comparable (Fig. 4). After 122 h of cultivation, the total amino acid content was approximately 8.5 g/L, with Lys predominating (from 6.8 to 7.2 g/L), followed by glycine (Gly ≤ 0.2 g/L). In contrast, at the low DO level of 0 ppm, the concentration of total amino acid reduced to 3.9 g/L (Table 1), with alanine (Ala) predominating, followed by Lys and valine (Val) (1.5, 1.3, and 0.9 g/L, respectively). Under oxygen limitation, a significant amount of organic acid was accumulated. After 122 h of cultivation, the concentrations of succinate, acetate, and lactate were 4.4, 4.9, and 1.5 g/L, respectively (Fig. 4). In contrast, at DO levels ≥ 1.3 ppm, the concentration of acetate was reduced by 73% (≤ 1.3 g/L), and succinate and lactate were scarcely observed. The total amount of metabolites produced was comparable in the range of 8.18–8.88 g/L at DO levels ≥ 1.3 ppm, while the total amount increased by about two-fold (15.98 g/L) at DO = 1 ppm (Table 1). These results suggest that glucose metabolism drastically shifted at DO levels < 1.3 ppm to produce organic acids instead of amino acids, and that reduced formation of Lys can increase the usage of carbon available for 3,4-AHBA formation.

**Table 1:** Effect of dissolved oxygen (DO) on fermentation products and cell growth

	DO (ppm)				
	0	1.3	2.6	5.3	8.0
3,4-AHBA (g/L)^a^	1.30 ± 0.04	0.61 ± 0.03	0.30 ± 0.02	0.37 ± 0.01	0.30 ± 0.01
Total amino acids (g/L)^a^	3.86 ± 0.12	7.03 ± 0.08	7.47 ± 0.01	6.56 ± 0.11	7.14 ± 0.01
Acetate (g/L)^a^	4.85 ± 0.14	1.14 ± 0.01	1.04 ± 0.01	1.13 ± 0.01	1.31 ± 0.09
Lactate (g/L)^a^	1.50 ± 0.11	0.10 ± 0.01	0 ± 0	0.07 ± 0.01	0.06 ± 0.01
Succinate (g/L)^a^	4.38 ± 0.36	0 ± 0	0 ± 0	0 ± 0	0 ± 0
DCW (g/L)^a,b^	3.59 ± 0.11	5.83 ± 0.08	7.63 ± 0.02	7.59 ± 0.20	7.10 ± 0.35
Total (g/L)^c^	19.48 (15.98)	14.71 (8.88)	16.44 (8.81)	15.72 (8.18)	15.91 (8.81)

^a^Each value was determined after 122 h of cultivation during 3-amino-4-hydroxybenzoic acid (3,4-AHBA) production by recombinant *C. glutamicum* strain KT01. Data are presented as the mean ± standard deviation calculated from the results of duplicate independent experiments. Strain KT01 was grown under aerobic conditions until the late log phase in brain heart infusion medium, and the cultures were then inoculated at an initial OD_600_ of 0.2 into modified mineral salt CGX II medium containing glucose (40 g/L) as the sole carbon source

^b^Dry cell weight (DCW) was determined by the following equation: an OD_600_ of 1.0 corresponded to 0.39 mg dry weight cell per milliliter

^c^Values in parenthesis indicate the total amounts of the produced metabolites

### Metabolic profiles at three different DO concentrations

**Fig. 5:**
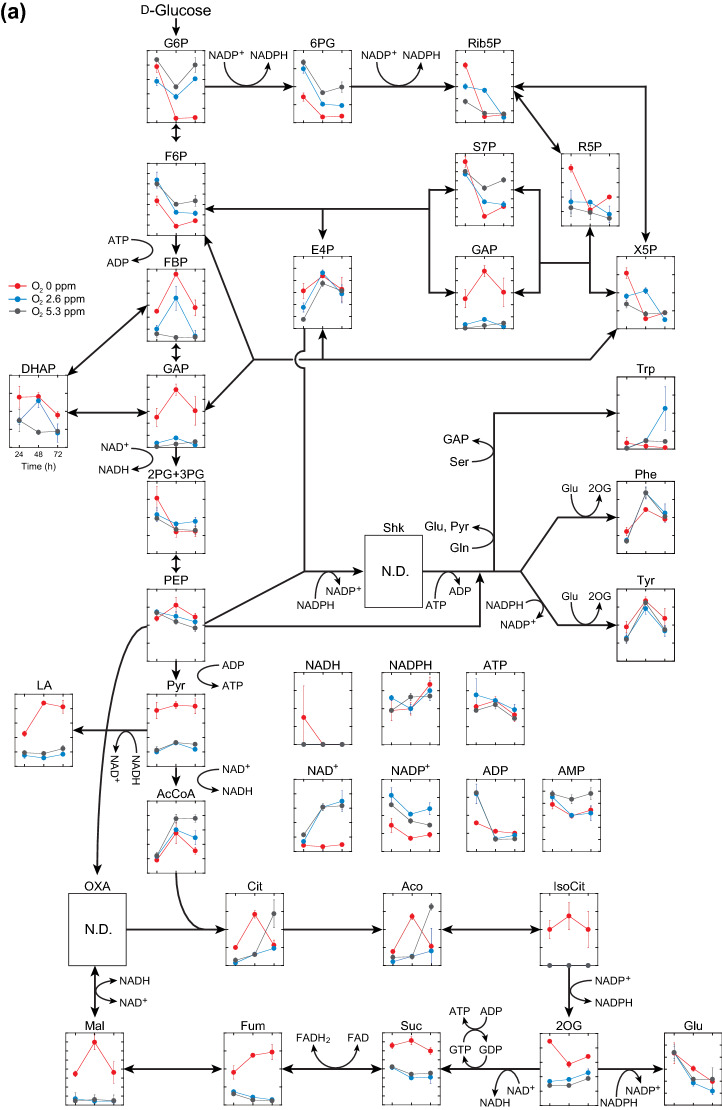

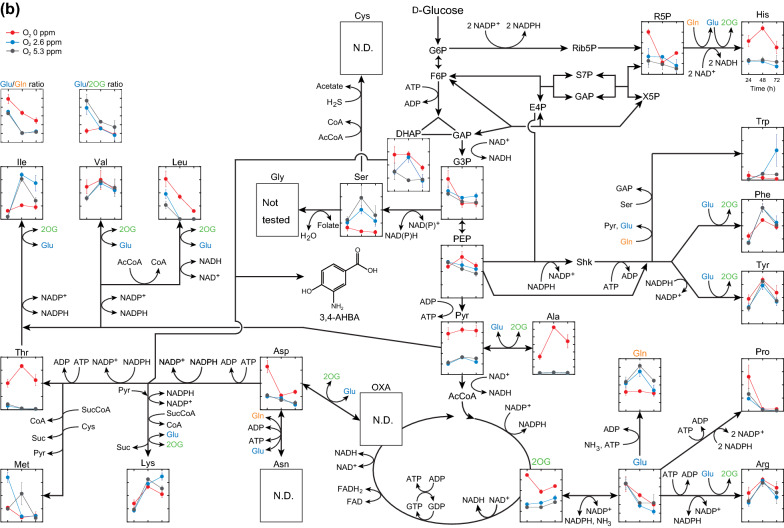
Time course metabolomics of in the central metabolic pathway (**a**) and amino acid metabolism (**b**). Relative abundances of metabolic intermediates in recombinant *C. glutamicum* strain KT01 during 3-amino-4-hydroxybenzoic acid (3,4-AHBA) production under controlled dissolved oxygen (DO) levels at 0, 2.6, and 5.3 ppm using the DO-stat program in synthetic CGX II medium containing d-glucose as the sole carbon source are shown. The X-axis indicates cultivation time (h). For the profiling experiment, strain KT01 was grown under aerobic conditions until the late log phase in brain heart infusion medium, and the culture was then inoculated to an initial OD600 of 0.2 into CGX II medium containing d-glucose as the sole carbon source (40 g/L). The cells were harvested at 24, 48, and 72 h of cultivation and subsequently subjected to metabolome analysis. Data are presented as average ± standard deviation calculated from the results of triplicate individual experiments. *N.D.* not determined below the detection limit

To investigate the change in the metabolic state under oxygen limitation, the metabolic profiles of *C. glutamicum* cells during 3,4-AHBA production were compared at three different DO levels (0, 2.6, and 5.3 ppm). In the central metabolic pathway, most metabolic intermediates showed similar profiles at DO levels of 2.6 and 5.3 ppm, while some exhibited specific profiles under oxygen limitation (DO = 0 ppm) (Fig. 5a). For instance, in the glycolytic pathway, reduced levels of fructose-6-phosphate and significantly increased levels of fructose-1,6-bisphosphate (FBP), glyceraldehyde-3-phosphate (GAP), and pyruvate were observed at a DO level of 0 ppm, as compared to DO ≥ 2.6 ppm (Fig. 5a). In addition, the level of DHAP, a precursor for 3,4-AHBA synthesis, was markedly increased under oxygen limitation. Acetyl-CoA (AcCoA) levels were reduced with decreasing DO levels, while significantly increased levels of pyruvate, and its derivative of lactate, were observed under oxygen limitation (DO = 0 ppm). In the TCA cycle, levels of five sequential metabolic intermediates (*iso*-citrate, 2-oxoglutarate, succinate, fumarate, and malate) were significantly increased under oxygen limitation (DO = 0 ppm). In oxidative PPP, the levels of most metabolic intermediates gradually reduced with time, and the level of 6-phospho-d-glucono-1,5-lactone (6PG) was significantly reduced at lower DO levels (Fig. 5a). In cofactor metabolism, levels of both NAD^+^ and NADP^+^ were significantly lower under oxygen limitation (DO = 0 ppm) compared to those at DO levels ≥ 2.6 ppm. Conversely, the levels of adenosine 5ʹ-triphosphate (ATP) and adenosine 5ʹ-diphosphate (ADP) were not significantly different.

In contrast to the central metabolic pathway, DO levels had limited effects on the intracellular levels of amino acids (Fig. 5b). However, markedly reduced levels of glutamine were observed, whereas glutamate levels were relatively high at a DO of 0 ppm, resulting in a significantly increased ratio of glutamate/glutamine under oxygen limitation, as compared to DO levels ≥ 2.6 ppm. Under oxygen limitation (DO = 0 ppm), significantly increased levels of alanine and lactate, both pyruvate derivatives, were observed. For amino acids derived from aspartate (Asp), which serves as a precursor for 3,4-AHBA synthesis, levels of Asp were increased markedly and those of threonine were significantly increased at DO = 0 ppm, while Lys levels were comparable at the three DO levels (Fig. 5b). Under all conditions, leucine levels monotonically decreased as time progressed due to the auxotrophy for leucine of the host strain ATCC 21799 [24]. These results suggest that glucose metabolism, including amino acid synthesis, drastically changes under oxygen limitation at DO levels < 2.6 ppm during 3,4-AHBA production.

### Metabolic engineering for enhanced 3,4-AHBA production

The host strain ATCC 21799 was metabolically engineered to design a metabolic pathway tailored for oxygen limitation during 3,4-AHBA production. To use excess pyruvate under oxygen limitation for 3,4-AHBA production, four biosynthetic pathways of by-products were selected for analysis based on metabolome data (Fig. 5). To eliminate or reduce by-products of acetate, lactate, succinate, or alanine, the following were individually or collectively inactivated in the wild-type strain by gene disruption (Fig. 1): pyruvate dehydrogenase (encoded by *pqo*), succinyl-CoA:acetate CoA-transferase (encoded by *cat*), phosphate acetyltransferase (encoded by *pta*), and acetate kinase (encoded by *ackA*) for acetate synthesis, lactate dehydrogenase (encoded by *ldh*) for lactate synthesis, malate dehydrogenase (encoded by *mdh*) for succinate synthesis, and two aminotransferases (encoded by *alaT* and *avtA*) for alanine synthesis. A single Δ*ldh* mutant (HKC5021) and a double Δ*alaT*Δ*avtA* mutant (HKC5044) were developed to inactivate the formation of lactate and alanine, respectively (Table 2). To reduce the formation of acetate and succinate, a triple Δ*pqo*Δ*cat*Δ*pta-ackA* mutant (HKC5050) and single Δ*mdh* mutant (HKC5053) were also developed. These mutants were transformed by introducing the plasmid pCAC*griHI*, and the resulting transformants were selected based on chloramphenicol resistance and 3,4-AHBA from glucose (Table 2).

**Table 2:** Strains and plasmids used in this study

Name	Relevant characteristics	Reference or source
Strain		
*Escherichia coli* JM109	*recA1 endA1 gyrA96 thi hsdR17*(r_K_^–^ m_K_^+^) *e*14^–^ (*mcrA*) *supE44 relA1* Δ(*lac-proAB*)/F’ [*traD36 proAB*^+^ *lacI*^q^ *lacZ*ΔM15]	Takara Bio
*Corynebacterium glutamicum* ATCC 21799	l-Lysine producer resistant to S-2-aminoethyl-l-cysteine, a lysine analog	ATCC
*C. glutamicum* strain KT01	*C. glutamicum* ATCC 21799 bearing pCAC*griHI*	Kawaguchi et al. [11]
*C. glutamicum* strain HKC5021	Markerless mutant, Δ*ldh* (KaCgl_14550) of strain ATCC 21799	This study
*C. glutamicum* strain HKC5044	Markerless double mutant, Δ*alaT* Δ*avtA* (KaCgl_13940 and KaCgl_11440, respectively) of strain ATCC 21799	This study
*C. glutamicum* strain HKC5050	Markerless triple mutant, Δ*pqo*Δ*cat* Δ*pta_ackA* (KaCgl_11560, KaCgl_11130, KaCgl_13020 and KaCgl_13010, respectively) of strain ATCC 21799	This study
*C. glutamicum* strain HKC5053	Markerless mutant of Δ*mdh* (KaCgl_08800) of strain ATCC 21799	This study
*C. glutamicum* strain HKC5037	HKC5021 bearing pCAC*griHI*	This study
*C. glutamicum* strain HKC5051	HKC5044 bearing pCAC*griHI*	This study
*C. glutamicum* strain HKC5052	HKC5050 bearing pCAC*griHI*	This study
*C. glutamicum* strain HKC5057	HKC5053 bearing pCAC*griHI*	This study
Plasmid		
pCAC*griHI*	Cm^r^; *E. coli–Corynebacterium* sp. shuttle vector harboring *griH* and *griI* genes derived from *Streptomyces griseus* for 3,4-AHBA biosynthesis	Kawaguchi et al. [11]
pK19mobsacB	Kan^r^, mobilizable *E. coli* vector for the construction of insertion and deletion mutants of *C. glutamicum* (*oriV*, *sacB*, *lacZ*α)	ATCC
pK19mobsac-Δ*pta-ackAldh*	Kan^r^, pK19mobsacB with the deletion construct for *pta-ackA* (KaCgl_13010 and KaCgl_13020) of *C. glutamicum* ATCC 21799	This study
pK19mobsac-Δ*alaT*	Kan^r^, pK19mobsacB with the deletion construct for *alaT* (KaCgl_13940) of *C. glutamicum* ATCC 21799	This study
pK19mobsac-Δ*avtA*	Kan^r^, pK19mobsacB with the deletion construct for *avtA* (KaCgl_11440) of *C. glutamicum* ATCC 21799	This study
pK19mobsac-Δ*cat*	Kan^r^, pK19mobsacB with the deletion construct for *cat* (KaCgl_11130) of *C. glutamicum* ATCC 21799	This study
pK19mobsac-Δ*ldh*	Kan^r^, pK19mobsacB with the deletion construct for *ldh* (KaCgl_14550) of *C. glutamicum* ATCC 21799	This study
pK19mobsac-Δ*mdh*	Kan^r^, pK19mobsacB with the deletion construct for *mdh* (KaCgl_08800) of *C. glutamicum* ATCC 21799	This study
pK19mobsac-Δ*pqo*	Kan^r^, pK19mobsacB with the deletion construct for *pqo* (KaCgl_29450) of *C. glutamicum* ATCC 21799	This study

Using metabolically engineered strains of *C. glutamicum*, the specific productivity of 3,4-AHBA was compared to that of the parent strain. Compared to the parent strain KT01, the specific productivity of 3,4-AHBA was more than double in the Δ*ldh* (HKC5037) and Δ*pqo*Δ*cat*Δ*pta*-*ackA* (HKC5052) mutants, whereas the cell growth of these strains was significantly reduced (Table 3). A double Δ*alaT*Δ*avtA* mutant (HKC5051), which had the auxotrophy for alanine, showed a 1.2-fold higher specific productivity of 3,4-AHBA than the parent strain with reduced cell growth. In contrast, the specific productivity of 3,4-AHBA was reduced by 90% in the Δ*mdh* mutant (HKC5057), with significantly reduced cell growth, as compared to the parent strain KT01. During 3,4-AHBA production, the total amount of by-products formed was significantly lower in all of the metabolically engineered strains than in the parent strain KT01; in particular, the accumulation of organic acids (acetate, lactate, and succinate) was significantly reduced (Table 3). In addition, Lys formation was comparable in Δ*ldh* (HKC5037) but significantly increased in the remaining three mutants, Δ*pqo*Δ*cat*Δ*pta*-*ackA* (HKC5052), Δ*alaT*Δ*avtA* (HKC5051), and Δ*mdh* (HKC5057), as compared to that in the parent strain.

**Table 3:** 3-amino-4-hydroxybenzoic acid (3,4-AHBA) production from glucose by metabolically engineered strains of *C. glutamicum*

	Parent (KT01)	Δ*ldh* (HKC5037)	Δ*pqo*Δ*cat*Δ*pta*-*ackA* (HKC5052)	Δ*alaT*Δ*avtA*^c^ (HKC5051)	Δ*mdh* (HKC5057)
3,4-AHBA (g/L)^a^	1.140 ± 0.085	1.950 ± 0.283	0.906 ± 0.199	1.190 ± 0.046	0.030 ± 0.017
Cell growth (g DCW/L)^a^	7.2 ± 0.3	3.5 ± 0.5	2.9 ± 0.6	6.6 ± 0.9	2.8 ± 1.1
Specific productivity (mg/h/g DCW)^a^	157.9 ± 5.7	561.7 ± 1.2	314.8 ± 31.5	183.3 ± 24.0	14.6 ± 15.1
Relative productivity (%)^b^	100 ± 4	365 ± 1	205 ± 20	119 ± 16	10 ± 10
By-product					
Lactate (g/L)^a^	1.12 ± 0.35	N.D.	N.D.	0.54 ± 0.10	0.02 ± 0.01
Acetate (g/L)^a^	2.88 ± 0.81	0.90 ± 0.49	0.81 ± 0.01	1.63 ± 0.22	N.D.
Succinate (g/L)^a^	3.39 ± 0.28	N.D.	N.D.	0.39 ± 0.39	N.D.
Ala (g/L)^a^	1.51 ± 0.07	0.54 ± 0.02	0.63 ± 0.02	0.21 ± 0.04	N.D.
Val (g/L)^a^	0.92 ± 0.01	0.69 ± 0.07	0.66 ± 0.00	0.60 ± 0.11	N.D.
Lys (g/L)^a^	1.30 ± 0.06	1.60 ± 0.18	2.86 ± 0.07	4.06 ± 0.08	2.55 ± 0.49
Total amounts of by-product (g/L)	11.12	3.73	4.96	7.43	2.57

*N.D.* not detected

^a^Values were determined after 100 h of cultivation. Data are presented as the mean ± standard deviation calculated from the results of duplicate independent experiments. Specific productivity was determined based on dry cell weight (DCW) after 100 h of cultivation. All strains were grown under aerobic conditions until the late log phase in brain heart infusion medium, and the cultures were then inoculated at an initial OD_600_ of 0.2 into modified mineral salt CGX II medium containing glucose (40 g/L) as the sole carbon source

^b^Relative productivity was determined based on the specific productivity of 3,4-AHBA

^c^For 3,4-AHBA production, alanine (final 100 mg/L) was added to CGX II medium because this mutant required alanine for cell growth

### Improved 3,4-AHBA production by a rationally designed strain of *C. glutamicum*

**Fig. 6:**
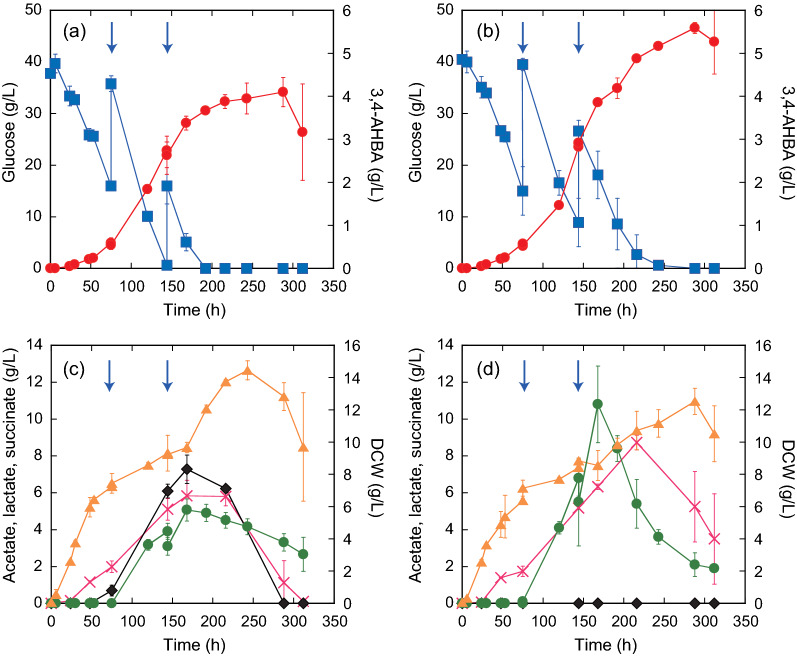
3-Amino-4-hydroxybenzoic acid (3,4-AHBA) production in glucose fed-batch cultures using two metabolically engineered *C. glutamicum* strains. For the profiling experiment, either parent strain KT01 (**a** and **c**) or strain HKC5037 (Δ*ldh* mutant) (**b** and **d**) was grown under aerobic conditions until the late log phase in brain heart infusion medium, and the culture was then inoculated to an initial OD_600_ of 0.2 into CGX II medium containing glucose (40 g/L) as the sole carbon sources. Glucose was fed twice as indicated in arrows to increase glucose concentration of 20 g/L. The concentrations of 3,4-AHBA (red circles), glucose (squares), acetate (crosses), lactate (diamonds), succinate (green circles), and cells (triangles) are shown. Data are presented as average ± standard deviation calculated from the results of duplicate individual experiments

Based on the comparative results shown in Table 3, the Δ*ldh* mutant (HKC5037) was selected for improved 3,4-AHBA production with the highest specific productivity, and the 3,4-AHBA production was compared with the parent strain KT01 in glucose fed-batch fermentation. The fed-batch fermentation was conducted with constant agitation at 200 rpm to maintain the DO level at 0 ppm after 24 h of cultivation. After 228 h of cultivation, 5.6 g/L of 3,4-AHBA was produced by the metabolically engineered *C. glutamicum* HKC5037 strain (Δ*ldh* mutant), whereas the parent strain KT01 produced 4.2 g/L of 3,4-AHBA (Fig. 6a, b). Within the first feed of glucose (75 h of cultivation), the glucose consumption rate and cell growth of the HKC5037 strain were comparable with that of the parent strain. This was attained by including urea as the nitrogen source in the CGXII medium to improve the poor growth of the HKC5037 strain under oxygen limitation, as shown in Table 3 and Additional file 1: Fig. S1. The *ldh* HKC5037 mutant completely lost its ability to produce lactate, while the transient accumulation of succinate after 168 h of cultivation increased by more than two folds than that in the parent strain (10.8 and 5.1 g/L, respectively) (Fig. 6c, d). In addition, the acetate concentration after 216 h of cultivation was 1.5-fold higher in the culture with the HKC5037 strain than the parent strain (8.7 and 5.8 g/L, respectively), although the production rate was comparable. Consequently, 3,4-AHBA yield after 288 h of cultivation was 1.4-fold higher by the rationally designed strain HKC5037 than that in the parent strain (0.070 and 0.053 g of 3,4-AHBA/g of glucose, respectively).

## Discussion

The pathway of 3,4-AHBA synthesis is specific to the actinomycete *Streptomyces griseus*. In this strain, the aromatic ring is formed through a two-step reaction from primary metabolites of DHAP in glycolysis and l-aspartate-4-semialdehyde as the Asp metabolite by the aldol condensation reaction [10], whereas most aromatic compounds in bacteria are predominantly synthesized in a multistep reaction via the shikimate pathway, which is subjected to multiple feedback regulations [1, 9, 25]. *C. glutamicum* strain ATCC 21799 has a gene for a feedback-resistant aspartate kinase in its chromosome [26], and produces a large amount of Lys under aerobic conditions (50% DO saturation) [27]. In contrast, the present study demonstrated that the carbon flux of l-aspartate-4-semialdehyde shifted toward 3,4-AHBA instead of Lys under oxygen limitation at a DO level of 0%.

Oxygen limitation changes the redox state of microbial cells [12, 28]. For the production of aromatic compounds, the DO level is generally controlled at ≥ 20% of saturated oxygen, such as the production of phenylalanine (Phe) [29] by *C. glutamicum* and the production of tryptophan (Trp) [30], shikimate [19], and Tyr [31] by *E. coli*. In contrast, the specific productivity of 3,4-AHBA increased with reduced DO levels, reaching a maximum concentration at a DO concentration of 0 ppm (Fig. 2). Apparently, glucose metabolism was shifted at a threshold of DO level between 0 and 1.3 ppm (corresponding to 17% O_2_ saturation). Consequently, the concentration of Lys, a predominant amino acid, was reduced by 81%, and the amount of organic acids conversely increased 8.3-fold under DO = 0 ppm, as compared to the aerobic conditions (Fig. 4). In contrast, the composition and concentration of extracellular amino acids and organic acids were comparable at DO levels ≥ 1.3 ppm. Even at a DO concentration of 1.3 ppm, the specific productivity of 3,4-AHBA was reduced by 49% as compared to DO = 0 ppm (Fig. 2). Compared to 3,4-AHBA biosynthesis, Lys biosynthesis requires more NADPH (4 mol of NADPH for 1 mol of Lys) [16], which is predominantly supplied from the oxidative PPP by aerobic metabolism [15, 20, 32] (Fig. 1).

In the present study, intracellular levels of both 6PG in PPP and NADP^+^, as the cofactor for Lys biosynthesis, were significantly lower at a DO level of 0 ppm than at ≥ 2.6 ppm (Fig. 5a). The reduced 6PG level under oxygen limitation conditions implies reduced flux through the oxidative PPP at a DO level of 0 ppm. This is supported by a previous study that showed that the flux from glycolysis to PPP decreased with reduced DO levels [33]. However, the NADPH levels were comparable between high and low DO levels, while the NADP^+^ levels were reduced under low DO levels (Fig. 5a). In *C. glutamicum*, malic enzyme, which reduces NADP^+^ to NADPH coupled with the oxidative decarboxylation of malate to pyruvate (Fig. 1), was induced under oxygen limitation [23]. The enhanced malic enzyme activity can compensate NADPH supply for reduced PPP flux under oxygen limitation [34]. Nevertheless, NADPH supply under oxygen limitation was likely to be limited for amino acid biosynthesis (Table 1), which requires NADPH as a cofactor [15] (Fig. 1). In addition, oxygen limitation induces the expression of genes responsible for organic acid production, which in turn oxidizes NADH to regenerate NAD^+^, instead of aerobic respiration, for ATP generation [22, 23]. However, NAD^+^ generation was also limited under oxygen limitation (Fig. 5a), resulting in reduced cell growth (Table 1). Consequently, oxygen limitation (DO = 0 ppm) increased the total amounts of metabolites produced while reducing cell growth and shifted glucose metabolism to produce organic acids instead of amino acids, as compared to DO ≥ 1.3 ppm (Table 1). These results suggest that a strict rate-limiting oxygen supply is critical for the enhanced production of 3,4-AHBA by recombinant *C. glutamicum* to control the redox state.

In addition to the enhanced 3,4-AHBA metabolism, oxygen limitation has another advantage for increased 3,4-AHBA titer. A previous study reported that 3,4-AHBA was non-enzymatically oxidized to form a yellow pigment under aerobic conditions [10]. This suggests that oxygen limitation (DO = 0 ppm) can attenuate the oxidation of the 3,4-AHBA produced, occurring under aerobic conditions (DO ≧ 2.6 ppm). To the best of our knowledge, this is the first report on the enhanced production of aromatic compounds under strictly oxygen-limited conditions at a DO concentration of 0 ppm.

Controlling the oxygen supply and thereby recycling cofactor NAD(P)^+^ is one strategy to optimize the performance of fermentation. However, knowledge regarding the comparative profiles between aerobic and oxygen-limited conditions remains limited [12]. Comparable metabolome analysis would provide insights into the change in metabolic status in response to DO concentration in both sugar and amino acid metabolism. Besides 3,4-AHBA production, metabolic profiles were altered at a DO threshold level between 0 ppm and 2.6 ppm. At a DO level of 0 ppm, intracellular concentrations of NAD^+^ and NADP^+^ were significantly lower than those under aerobic conditions (Fig. 5a). Under oxygen limitation, the intracellular concentration of NAD^+^ was significantly reduced due to the shortage of NAD^+^ recycling coupled with aerobic respiration [35], although some NAD^+^ can be regenerated by lactate dehydrogenase and malate dehydrogenase under oxygen limitation [36]. Moreover, the limited NAD^+^ recycling blocked all redox reactions requiring NAD^+^ as a cofactor and consequently accumulated pyruvate and GAP (Fig. 5a). At a DO level of 0 ppm, a reduced NAD^+^/NADH ratio, associated with the downregulation of the NADH dehydrogenase gene, was also observed in *C. glutamicum* cells in a previous study [21].

The accumulation of pyruvate resulted in increased intracellular and extracellular lactate levels and alanine at a DO level of 0 ppm (Figs. 4, 5a, b). Under oxygen limitations, intracellular levels of Asp was markedly increased, and that of threonine (Thr) was significantly increased, while Lys levels were unaffected (Fig. 5b), suggesting that the synthesis of amino acids, including Lys, is suppressed due to limited NADPH supply [37] and that the surplus l-aspartate-4-semialdehyde can be used for 3,4-AHBA synthesis. Under oxygen limitation, upregulation of triosephosphate isomerase, which catalyzes the reversible conversion of GAP and DHAP, was observed in previous studies [21, 23], which allows an increase in DHAP supply for 3,4-AHBA biosynthesis (Fig. 5a). In previous studies, genome-based flux balance analysis was adopted to predict and measure the operation and regulation of metabolic networks [38]. In contrast, the present metabolomics analysis revealed metabolic shifts in both the central metabolic pathway and amino acid metabolism, particularly in pyruvate-derived amino acids under strict oxygen limitation (DO = 0 ppm), although intracellular concentrations of amino acids were comparable between high and low DO levels [22].

Comparative analysis of both extracellular and intracellular concentrations of metabolites between aerobic and oxygen-limited conditions pointed to a metabolic shift in response to DO concentration (Figs. 4, 5a, b). Based on these results, the metabolic pathway of the 3,4-AHBA-producing strain KT01 was rationally engineered to perform at its full potential under oxygen limitation (Fig. 1, Table 3). The *mdh* mutant showed poor cell growth and produced negligible levels of 3,4-AHBA under oxygen limitation (Table 3). The *mdh* gene encoding malate dehydrogenase plays an important role in NAD^+^ recycling under anaerobic conditions [36], and the *mdh* mutant showed a significantly reduced glucose consumption rate [32]. These results suggest that *mdh* disruption remarkably diminishes the capability of NAD^+^ recycling at a DO concentration of 0 ppm. In addition, the inability to convert oxaloacetate (OXA) into malate blocked succinate production, but the excess OXA was likely to be used for enhanced Lys production (Table 3). In *C. glutamicum*, two aminotransferases encoded by *alaT* and *avtA* are exclusively responsible for alanine synthesis [39]. The specific productivity of 3,4-AHBA in the double mutant Δ*alaT*Δ*avtA* was comparable to that of the parent strain (Table 3). If the alanine biosynthetic pathway is blocked, the precursors of pyruvate and glutamate can be used for the synthesis of other pyruvate-derived metabolites [40]. Consequently, this mutant showed the highest productivity for total by-products, especially Lys, among the four metabolically engineered strains (Fig. 4), suggesting a limited effect of the Δ*alaT*Δ*avtA* mutant on enhanced 3,4-AHBA production. The Δ*ldh* mutant and Δ*pqo*Δ*cat*Δ*pta*-*ackA* triple mutant were designed to lose lactate productivity [41] or reduce acetate productivity [42], respectively. These two strains showed more than two times increased specific productivity of 3,4-AHBA compared to that of the parent strain KT01 (Table 3). Compared to the Δ*ldh* mutant, the Δ*pqo*Δ*cat*Δ*pta*-*ackA* triple mutant showed 1.8-fold higher Lys productivity, while other metabolites were produced at comparable levels. In contrast, the Δ*ldh* mutant showed the lowest productivity of Lys, while the levels of other pyruvate-derived metabolites, such as acetate, Ala, and Val, were comparable to that of the Δ*pqo*Δ*cat*Δ*pta*-*ackA* triple mutant. These results suggest that the lower production of other by-products allows the Δ*ldh* mutant to improve its specific productivity of 3,4-AHBA.

## Conclusions

In the present study, we demonstrated that both extracellular and intracellular metabolic profiles were constant at DO ≥ 2.6 ppm, corresponding to ≥ 33% saturated oxygen at 26 °C; however, this profile changed remarkably under oxygen limitation (DO = 0 ppm). Consequently, we revealed that 3,4-AHBA production reached the maximum level at a DO of 0 ppm, whereas the fermentation of other aromatic compounds was optimized at a DO of ≥ 10% saturated oxygen. To the best of our knowledge, this is the first study to report a comparative measurement of metabolic intermediates instead of a conventional approach using genome-based flux balance analysis to optimize DO conditions for microbial production. The metabolomics analysis revealed the points at which the metabolism shifted in response to DO concentration, particularly under oxygen limitation (DO = 0 ppm). Under oxygen limitation, we observed significantly reduced NAD(P)^+^ levels and altered levels of relevant metabolic intermediates requiring NAD(P)^+^ as cofactors. These comprehensive data regarding metabolic states in response to DO provide insights into the oxygen supply during fermentation and the rational design of metabolic pathways for improved production of related amino acids and their derivatives.

## Methods

### Bacterial strains and media

The bacterial strains and plasmids used in this study are listed in Table 2. For genetic manipulations, *E. coli* strains were grown at 37 °C in Luria–Bertani medium [43]. For the aerobic growth of *C. glutamicum*, the wild-type and recombinant strains were grown at 30 °C to the late log phase in brain heart infusion (BHI) broth (BD Biosciences, Franklin Lakes, NJ, USA), unless indicated otherwise. For AHBA production, modified mineral salt CGXII medium [44] containing glucose as the carbon source supplemented with leucine (100 mg·L^−1^) and pantothenic acid (100 mg·L^−1^) without urea was used. When appropriate, media were supplemented with 5 µg·mL^−1^ and 50 µg·mL^−1^ chloramphenicol for *C. glutamicum* and *E. coli*, respectively.

### DNA manipulation

All restriction endonucleases were purchased from New England Biolabs (Ipswich, MA, USA). PrimeSTAR Max DNA Polymerase (Takara Bio, Kusatsu, Japan) was used for PCR to amplify DNA fragments, according to the manufacturer’s instructions. PCR fragments were purified using a QIAquick PCR Purification Kit (Qiagen, Hilden, Germany). Plasmids were developed using an In-Fusion HD Cloning Kit (Takara Bio) for seamless ligation-independent cloning of PCR fragments, according to the manufacturer’s instructions. Electroporation was used to transform *C. glutamicum*, as previously described [45], whereas *E. coli* was transformed using the CaCl_2_ procedure [43]. Plasmid DNA was isolated from *E. coli* as previously described [46].

### Construction of metabolically engineered *C. glutamicum* strains

*C. glutamicum* strain ATCC 21799 was metabolically engineered to improve 3,4-AHBA production based on its whole genome sequence (DDBJ/ENA/GenBank accession number AP022856.1) [47]. Deletion mutants of *C. glutamicum* ATCC 21799 were developed using a suicide vector system followed by a two-step homologous recombination procedure, as previously described [48]. For *pta-ackA*, plasmid pK19mobsac-Δ*pta-ackA* was constructed by PCR amplification of the gene with both flanking regions using the *C. glutamicum* ATCC 21799 genome as the template and oligonucleotide primer pairs 1, 2, 3, and 4 (Table 4), respectively. The PCR-amplified upstream and downstream fragments were integrated into *Pst*I-and *EcoRI-*digested plasmid pK19mobsacB (American Type Culture Collection, Manassas, VA, USA) by multiple-fragment cloning using In-Fusion, yielding the construct pK19mobsac-Δ*pta-ackA* (Table 2). Gene deletion was confirmed by PCR using oligonucleotide primers 1 and 4. Analysis of *C. glutamicum* transformants, selected on the basis of kanamycin sensitivity and sucrose resistance, ultimately yielded a markerless *pta-ackA* deletion mutant (21799Δ*pta-ackA*) (Table 2). Likewise, plasmids pK19mobsac-Δ*alaT*, pK19mobsac-Δ*avtAT*, pK19mobsac-Δ*ldh*, pK19mobsac-Δ*pqo*, pK19mobsac-Δ*cat*, and pK19mobsac-Δ*mdh* were generated (Table 2), for the transformation of *E. coli* JM109. Using the resulting plasmids, deletion mutants of 21799Δ*alaT*Δa*vtA* (strain HKC5044), 21799Δ*ldh* (strain HKC5021), 21799Δ*mdh* (strain HKC5053), and 21799Δ*pqo*Δ*cat* Δ*pta-ackA* (strain HKC5050) were constructed (Table 2). The deletion mutant was transformed by electroporation with the plasmid pCAC*griHI* to express *griH* and *griI* derived from *Streptomyces griseus*, which is responsible for 3,4-AHBA synthesis (Table 4) [11]. Transformants were selected by chloramphenicol resistance and were subsequently screened for their ability to produce 3,4-AHBA from glucose as the sole carbon source. A positive transformant was selected for further characterization (Table 2).

**Table 4:** Oligonucleotides used in this study

Name	Target gene	Sequence (5′–3′)	Cohesive ends^a^
Primer 1	*pta-ackA*	ACGGCCAGTGGAATTCTGCGTGAGATGAAGTAAGGC	*Eco*RI
Primer 2	*pta-ackA*	GGTGTTGGTGAAAATGCCCA	
Primer 3	*pta-ackA*	ATTTTCACCAACACCACGTGTTTCCTACACCGATG	
Primer 4	*pta-ackA*	ATGATTACCCAAGCTTGTCCGTGTCGGATTTCATCA	*Hin*dIII
Primer 5	*alaT*	ACGGCCAGTGGAATTCCTAGTCCGTTTTCGACGATG	*Eco*RI
Primer 6	*alaT*	ACAGCACGTCCTTCATCTTC	
Primer 7	*alaT*	TGAAGGACGTGCTGTACCCCAACGTGTACGAAATC	
Primer 8	*alaT*	ATGATTACCCAAGCTTAAGTTTCAGGCATAGGCAGG	*Hin*dIII
Primer 9	*avtA*	ACGGCCAGTGGAATTCTCCATGAGGTCAAGCATGTC	*Eco*RI
Primer 10	*avtA*	CCGATGATTCAGAGGAATGG	
Primer 11	*avtA*	CCTCTGAATCATCGGAGAGCGATCTCTGCTTCTTC	
Primer 12	*avtA*	ATGATTACCCAAGCTTTTGATGGGGAGACTGTGGTT	*Hin*dIII
Primer 13	*cat*	ACGGCCAGTGGAATTCCGTAAAGCGGAGTTTTAGGC	*Eco*RI
Primer 14	*cat*	ATCTCTGAGTACGGTTACGC	
Primer 15	*cat*	ACCGTACTCAGAGATAACAGGTCGATTGCGTAGTC	
Primer 16	*cat*	ATGATTACCCAAGCTTAGCAACGTTGGTTACACCAG	*Hin*dIII
Primer 17	*ldh*	ACGGCCAGTGGAATTCTGGGTTAATTCGCCGGTGATCAG	*Eco*RI
Primer 18	*ldh*	GGTTGATCAGTGCAGTATGCGTATG	
Primer 19	*ldh*	CTGCACTGATCAACCCACTGCTCCACGGTGAATACG	
Primer 20	*ldh*	ATGATTACCCAAGCTTGGCAAGGTCCATGCTGACG	*Hin*dIII
Primer 21	*mdh*	ACGGCCAGTGGAATTCAATGACAACGGCGTGGCTTC	*Eco*RI
Primer 22	*mdh*	TCTGCGGCAATTCCTTCCAC	
Primer 23	*mdh*	AGGAATTGCCGCAGAGGGATCTCCAGAAGTTTCAG	
Primer 24	*mdh*	ATGATTACCCAAGCTTTTTCCATCAATAGCAGGCGC	*Hin*dIII
Primer 25	*pqo*	ACGGCCAGTGGAATTCTGGTCGCATCTCATCGATTG	*Eco*RI
Primer 26	*pqo*	GCATATCCTGGACCTGTACT	
Primer 27	*pqo*	CAGGATATGCTTTCCAGGACCACAAGAAGC	
Primer 28	*pqo*	ATGATTACCCAAGCTTCTTGCGCCTGCAAAGTTTCT	*Hin*dIII

^a^The restriction site overhangs used in the cloning procedure are underlined

### 3,4-AHBA production by recombinant *C. glutamicum*

Recombinant *C. glutamicum* strains were grown aerobically to the late log phase in a 50 mL test tube containing BHI broth with constant agitation (180 rpm) for 18 h at 26 °C. The pre-culture was transferred to 90 mL of modified mineral salt CGXII medium containing glucose (40 g L^−1^ glucose) in a 200 mL jar fermentor Bio Jr.8 BJR-25NA1S-8 M (ABLE Co. & Biott Co., Tokyo, Japan) to obtain a cell concentration corresponding to an optical density at 600 nm (OD_600_) of 0.2. The cultivation conditions were as follows: temperature, 26 °C; pH, 7.0, maintained by the addition of ammonia; stirrer speed, 200 rpm; aeration with compressed air, 0.5 vvm, unless indicated otherwise. For dissolved oxygen (DO)-stat cultivation of 3,4-AHBA production, the DO-stat method was started after 24 h of cultivation to control DO levels (0, 1.3, 2.6, 5.3, and 8.0 ppm) by changing the agitation speed.

### Analytical procedures

Culture samples were centrifuged (15,000 × *g*, 4 °C, 10 min), and the concentrations of 3,4-AHBA, organic acids (acetate, lactate, and succinate), and glucose in the resulting supernatants were measured by high-performance liquid chromatography (HPLC), as previously described [11]. The concentrations of free amino acids in the resulting supernatants were determined with an ultra-high-performance liquid chromatograph (Nexera X2; Shimadzu, Kyoto, Japan) using an AccQ Tag Ultra Chemistry Kit for amino acid analysis (Waters, Milford, MA, USA), as previously described [49]. Cell mass was estimated by measuring the OD_600_ using a spectrophotometer (U-3010; Hitachi, Tokyo, Japan). An OD_600_ of 1.0 corresponded to 0.39 mg dry weight cell per milliliter [46].

Metabolome analysis of *C. glutamicum* cells was conducted as previously described [50]. Major metabolites of the central metabolic pathways (e.g., glycolysis, PPP, and tricarboxylic acid [TCA] cycle) were analyzed using an ion-pairing LC–MS/MS method [51]. Dried cell extracts were dissolved in 50 µL of MilliQ water for LC–MS/MS profiling and quantitation of 30 intracellular *C. glutamicum* metabolites. The following metabolites were analyzed: sugar phosphates (glucose-6-phosphate [G6P], fructose-6-phosphate [F6P], frucotose-1,6-bisphosphate [FBP], DHAP, glyceraldehyde-3-phosphate [GAP], 2- and 3-phosphoglycerate [2PG + 3PG], phosphoenolpyruvate [PEP], 6-Phospho-d-glucono-1,5-lactone [6PG], ribulose-5-phosphate [Rib5P], ribose-5-phosphate [R5P], X5P, erythrose-4-phosphate [E4P], and sedoheptulose-7-phosphate [S7P]); organic acids (aconitate, citrate [Cit], fumarate [Fum], isocitrate [IsoCit], malate [Mal], oxaloacetate [OXA], 2-oxoglutarate [AKG], pyruvate, and succinate [Suc]); nucleotides (adenosine di- [ADP] and triphosphate [ATP]); coenzymes (acetyl-CoA [AcCoA], oxidized and reduced nicotinamide adenine dinucleotide [NAD^+^ and NADH, respectively], oxidized and reduced nicotinamide adenine dinucleotide phosphate [NADP^+^ and NADPH, respectively]); and amino acids (alanine, [Ala], arginine [Arg], asparagine [Asn], aspartate [Asp], cysteine [Cys], glycine [Gly], glutamate [Glu], glutamine [Gln], histidine [His], isoleucine [Ile], leucine [Leu], lysine [Lys], methionine [Met], phenylalanine [Phe], proline [Pro], serine [Ser], threonine [Thr], tryptophan [Trp], tyrosine [Tyr], and valine [Val]). Metabolites were quantified as described previously [52] using an Agilent 1200 series MS and Agilent 6460 with Jet Stream Technology LC–MS/MS system (Agilent Technologies, Waldbronn, Germany) equipped with a Maestro C18 column (2.1 × 150 mm, 3-µm particle size; Shimadzu, Kyoto, Japan).

### Extraction of metabolic intermediates

For quantitative metabolomics, *C. glutamicum* cells were subjected to cold methanol quenching, as described previously [53], with slight modifications. A total of 15 mL of liquid culture at an OD_600_ of 2.0 was withdrawn from the medium and immediately sprayed into a 50 mL centrifugal tube (LMS Co., Ltd., Tokyo, Japan) containing double the volume of 40% (v/v) aqueous methanol at − 25 °C. After sampling, the content of each tube was immediately mixed by vortexing for 5 s to quench cellular metabolism and subsequently centrifuged (4000 × *g,* 5 min, − 9 °C). After decanting the supernatant, cell pellets were washed with 8 mL of 0.8% (w/v) NaCl at 4 °C and centrifuged again (4000 × *g*, 5 min, − 9 °C). After decanting the supernatant, the tubes containing the cell pellets were submerged directly into liquid nitrogen and stored at − 80 °C until metabolite extraction. For metabolite extraction, 3.0 mL of cold methanol (− 25 °C) containing ( +)-camphor-10-sulfonic acid (18 µg·L^−1^) was added to each tube as an internal standard for quantitative LC-MC/MS analysis. The tubes were vortexed for 30 s, and the resulting cell suspensions were incubated at − 30 °C for 1 h, after which 1.5 mL of the suspensions were transferred to 15 mL centrifuge tubes (LMS Co., Ltd.) containing 2.1 mL of chloroform and 1.5 mL of distilled water. These were then mixed by vortexing for 5 s. After centrifuging the resulting suspensions at 15,000 × *g* for 5 min at 4 °C, the upper phases were transferred to new tubes and dried under vacuum. The dried samples were stored at − 80 °C until the metabolite analysis was performed.

### Statistical analysis

Differences in sugar and metabolic intermediate concentrations and differences in cell density between the fermentation media were compared using the paired Student’s *t*-test. Statistical significance was set at *p* < 0.05.

## Supplementary Information

**Additional file 1: Fig. S1.** Cell growth of strain HKC5037 (Δ*ldh* mutant) under oxygen limitation (DO = 0 ppm) in the absence or presence of urea in modified CGX II medium. The strain HKC5037 was grown under aerobic conditions until the late log phase in brain heart infusion medium, and the culture was then inoculated at an initial OD600 of 0.2 into modified CGX II medium containing glucose (40 g/L) as the sole carbon source. During the cultivation, dissolved oxygen (DO) was controlled at 0 ppm using the DO-stat program.

## Data Availability

All the data analyzed during this study have been included in this published article and Additional data.

## Abbreviations

2PG + 3PG: 2- and 3-phosphoglycerate
3,4-AHBA: 3-Amino-4-hydroxybenzoic acid
6PG: 6-Phospho-d-glucono-1,5-lactone
AcCoA: Acetyl-CoA
ADP: Adenosine 5ʹ-diphosphate
AKG: 2-Oxoglutarate
Ala: Alanine
AMP: Adenosine 5’-monophosphate
Arg: Arginine
Asn: Asparagine
Asp: Aspartate
ATP: Adenosine 5ʹ-triphosphate
Cit: Citrate
Cys: Cysteine
DCW: Dry cell weight
DHAP: Dihydroxyacetone phosphate
DO: Dissolved oxygen
E4P: Erythrose-4-phosphate
F6P: Fructose-6-phosphate
FBP: Fructose-1,6-bisphosphate
Fum: Fumarate
G6P: Glucose-6-phosphate
GAP: Glyceraldehyde-3-phosphate
Gln: Glutamine
Glu: Glutamate
Gly: Glycine
Ile: Isoleucine
IsoCit: Isocitrate
His: Histidine
LA: Lactate
l-DOPA: l-Dihydroxyphenylalanine
Leu: Leucine
Mal: Malate
Met: Methionine
NAD^+^: Oxidized nicotinamide adenine dinucleotide
NADH: Reduced nicotinamide adenine dinucleotide
NADP^+^: Oxidized nicotinamide adenine dinucleotide phosphate
NADPH: Reduced nicotinamide adenine dinucleotide phosphate
OXA: Oxaloacetate
Phe: l-Phenylalanine
PEP: Phosphoenolpyruvate
PPP: Pentose phosphate pathway
Pro: Proline
Rib5P: Ribulose-5-phosphate
R5P: Ribose-5-phosphate
S7P: Sedoheptulose-7-phosphate
Ser: Serine
Suc: Succinate
TCA: Tricarboxylic acid
Thr: Threonine
Trp: l-Tryptophan
Tyr: l-Tyrosine
Val: Valine
X5P: Xylulose-5-phosphate
