# Supplementary material for: Enhanced production of γ-amino acid 3-amino-4-hydroxybenzoic acid by recombinant Corynebacterium glutamicum under oxygen limitation
Source: Microb Cell Fact. 2021 Dec 23;20:228. doi: 10.1186/s12934-021-01714-z (PMC8697445; doi:10.1186/s12934-021-01714-z)
Supplement: Supplementary file 1 — Additional file 1: Fig. S1. Cell growth of strain HKC5037 (Δldh mutant) under oxygen limitation (DO = 0 ppm) in the absence or presence of urea in modified CGX II medium. The strain HKC5037 was grown under aerobic conditions until the late log phase in brain heart infusion medium, and the culture was then inoculated at an initial OD600 of 0.2 into modified CGX II medium containing glucose (40 g/L) as the sole carbon source. During the cultivation, dissolved oxygen (DO) was controlled at 0 ppm using the DO-stat program. [file 12934_2021_1714_MOESM1_ESM.docx]

**Additional information**

Enhanced production of γ-amino acid 3-amino-4-hydroxybenzoic acid by recombinant *Corynebacterium glutamicum* under oxygen limitation

Hideo Kawaguchi^a,b^, Tomohisa Hasunuma^a,b^, Yasuo Ohnishi^c,d^, Takashi Sazuka^e^, Akihiko Kondo^a,b,f,g,^*, Chiaki Ogino^b,f^

^a^Graduate School of Science, Technology and Innovation, Kobe University, 1-1 Rokkodai, Nada, Kobe 657-8501, Japan

^b^Engineering Biology Research Center, Kobe University, 1-1 Rokkodai, Nada, Kobe 657-8501, Japan

^c^Department of Biotechnology, Graduate School of Agricultural and Life Sciences, The University of Tokyo, 1-1-1, Yayoi, Bunkyo, Tokyo 113-8657, Japan.

^d^Collaborative Research Institute for Innovative Microbiology, The University of Tokyo, Bunkyo, Tokyo 113-8657, Japan

^e^Bioscience and Biotechnology Center, Nagoya University, Furo, Chikusa, Nagoya 464-8601, Japan

^f^Department of Chemical Science and Engineering, Graduate School of Engineering, Kobe University, 1-1 Rokkodai, Nada, Kobe 657-8501, Japan

^g^Biomass Engineering Research Division, RIKEN, 1-7-22 Suehiro, Tsurumi, Yokohama, Kanagawa 230-0045, Japan

**Correspondence:** Akihiko Kondo

Graduate School of Science, Technology and Innovation, Kobe University

1-1 Rokkodai, Nada, Kobe 657-8501, Japan

Phone: +81-78-803-6196

Fax: +81-78-803-6192

E-mail: akondo@kobe-u.ac.jp

**Fig. S1** Cell growth of strain HKC5037 (Δ*ldh* mutant) under oxygen limitation (DO = 0 ppm) in the absence or presence of urea in modified CGX II medium. The strain HKC5037 was grown under aerobic conditions until the late log phase in brain heart infusion medium, and the culture was then inoculated at an initial OD_600_ of 0.2 into modified CGX II medium containing glucose (40 g/L) as the sole carbon source. During the cultivation, dissolved oxygen (DO) was controlled at 0 ppm using the DO-stat program
